# Muscle Afferent Receptors Engaged in Augmented Sympathetic Responsiveness in Peripheral Artery Disease

**DOI:** 10.3389/fphys.2012.00247

**Published:** 2012-07-10

**Authors:** Jianhua Li, Jihong Xing

**Affiliations:** ^1^Heart and Vascular Institute, Penn State University College of MedicineHershey, PA, USA

**Keywords:** static exercise, muscle afferents, sympathetic nerve activity, PAD, ASIC, P2X, TRPV1, NGF

## Abstract

The exercise pressor reflex (EPR) is a neural control mechanism responsible for the cardiovascular responses to exercise. As exercise is initiated, thin fiber muscle afferent nerves are activated by mechanical and metabolic stimuli arising in the contracting muscles. This leads to reflex increases in arterial blood pressure (BP) and heart rate primarily through activation of sympathetic nerve activity (SNA). Studies of humans and animals have indicated that the EPR is exaggerated in a number of cardiovascular diseases. For the last several years, studies have specifically employed a rodent model to examine the mechanisms at receptor and cellular levels by which responses of SNA and BP to static exercise are heightened in peripheral artery disease (PAD), one of the most common cardiovascular disorders. A rat model of this disease has well been established. Specifically, femoral artery occlusion is used to study intermittent claudication that is observed in human PAD. The receptors on thin fiber muscle afferents that are engaged in this disease include transient receptor potential vanilloid type 1 (TRPV1), purinergic P2X, and acid sensing ion channel (ASIC). The role played by nerve growth factor in regulating those sensory receptors in the processing of amplified EPR was also investigated. The purpose of this review is to focus on a theme namely that PAD accentuates autonomic reflex responses to exercise and further address regulatory mechanisms leading to abnormal sympathetic responsiveness. This review will present some of recent results in regard with several receptors in muscle sensory neurons in contribution to augmented autonomic reflex responses in PAD. Review of the findings from recent studies would lead to a better understanding in integrated processing of sympathetic nervous system in PAD.

## Introduction

**Figure 1:**
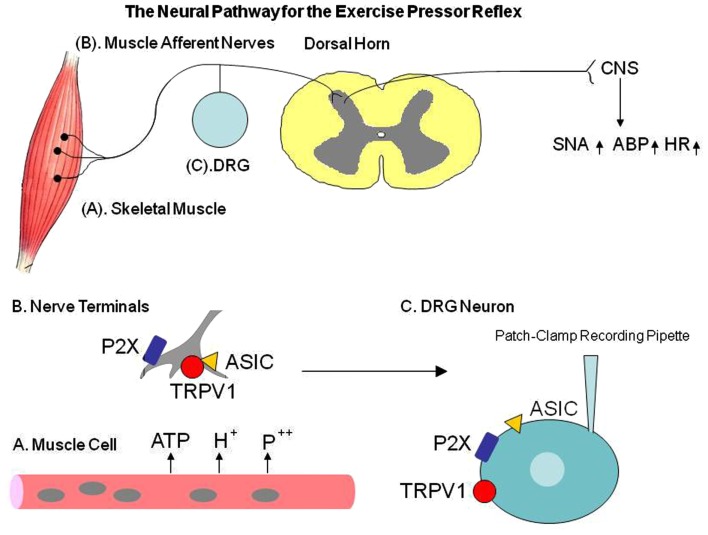
**The diagram describes the neural pathway for the exercise pressor reflex**. Interstitial ATP, H^+^, and phosphate are increased in exercising muscle. P2X, TRPV1, and ASIC mediate the muscle metabolite-induced reflex responses. In peripheral artery disease (PAD), those receptors in dorsal root ganglion (DRG) are altered, which likely leads to abnormal cardiovascular responses during exercise. Note that the whole cell patch clamp methods are used to characterize receptor responsiveness in the primary afferent neuron of control rats and PAD rats. Those receptors appear at the peripheral terminals and cell body of the DRG (sensory afferent neurons). The receptor activity in the DRG cell body can be used to reflect its effect in the nerve endings. TRPV1, transient receptor potential vanilloid type 1; ASIC, acid sensing ion channel; SNA, sympathetic nerve activity; ABP, arterial blood pressure; HR, heart rate.

During exercise, sympathetic nervous activity (SNA) increases and this leads to rises in blood pressure (BP) and heart rate (HR), myocardial contractility, and peripheral vasoconstriction (Victor et al., 1988; Sinoway et al., 1989). A basic mechanism termed the “Exercise Pressor Reflex” (EPR; Coote et al., 1971; McCloskey and Mitchell, 1972; Mitchell et al., 1977, 1983) is thought to contribute to sympathetic engagement during exercise (Figure 1). This autonomic reflex is initiated as thin fiber afferents arising from contracting skeletal muscle are engaged (McCloskey and Mitchell, 1972; Mitchell et al., 1983; Kaufman and Forster, 1996a). This system responds to mechanical deformation of the muscle afferents receptive field as well as to muscle by-products (Kaufman and Forster, 1996a). Group III afferents are predominantly mechanically sensitive (mechanoreceptor) and Group IV afferents are predominantly metabosensitive (metaboreceptor; Kaufman et al., 1984). When these receptors are stimulated, thin fiber muscle afferent nerves are engaged, cardiovascular nuclei in the brainstem are activated, SNA increases, and BP and HR rise (Mitchell et al., 1983). In addition, the sympathetic and cardiovascular responses to exercise are modulated by the “Central Command” (Goodwin et al., 1972; Waldrop et al., 1996), and the arterial baroreflex (Potts and Li, 1998; Fadel et al., 2001).

These reflex mechanisms found in healthy individuals are altered in cardiovascular diseases in the processing of muscle afferent signals via afferent nerves’ receptors (Li et al., 2004b; Sinoway and Li, 2005; Smith et al., 2006; Gao et al., 2007; Xing et al., 2008a; Liu et al., 2010, 2011; Tsuchimochi et al., 2010a). For example, as the EPR is activated in patients with peripheral arterial disease (PAD), increases in SNA, BP, and HR are exaggerated (Baccelli et al., 1999; Bakke et al., 2007). As noted, PAD caused by a restriction of the blood vessels in the lower limbs is typically popular in the older adults (Ouriel, 2001; Critchley and Capewell, 2003; Muir, 2009). The most common symptom of this disease is intermittent claudication, which frequently occurs during physical activity but is relieved promptly by rest (Rejeski et al., 2008). Thus, a rat model of femoral artery ligation that displays impaired reserve capacity of limb blood flow with exercise, has been employed to study PAD in humans (Waters et al., 2004). Using this rat model, a number of prior studies have further demonstrated that the SNA and pressor responses to static muscle contraction and stimulation of muscle metabolite receptors, i.e., capsaicin sensitive transient receptor potential vanilloid type 1 (TRPV1), purinergic P2X, and acid sensing ion channels (ASICs) are amplified in occluded rats as compared with control rats (Xing et al., 2008a; Liu et al., 2010, 2011; Tsuchimochi et al., 2010a; Figure 1). Nevertheless, the underlying mechanisms by which femoral occlusion augments responsiveness of SNA and BP to activation of muscle mechano- and metabo-sensitive afferents remain to be determined.

Prior studies demonstrated that femoral artery occlusion elevates the levels of nerve growth factor (NGF) in the hindlimb muscles and dorsal root ganglion (DRG) neurons of rats (Emanueli et al., 2002; Xing et al., 2009). NGF can induce expression of TRPV1, P2X3, and ASIC3 receptors in the DRG neurons (Ramer et al., 2001; Mamet et al., 2003). Therefore, NGF was infused into the hindlimb muscles using the osmotic minipump, and the role for NGF in regulating expression and response TRPV1, P2X3, and ASIC3 receptors was examined. In addition, NGF can change the neuronal phenotype such as capsaicin-insensitive sensory neurons (Hunter et al., 2000), which possibly alters afferent mediated response in the processing of sensory signals. Accordingly, the dual immunofluorescence techniques were employed to examine if femoral ligation and NGF can alter distribution of DRG neurons with the two thin fiber phenotypes: C-fiber and A-fiber. Also, whether femoral occlusion and NGF can selectively increase expression of ASIC3 in DRG neurons that project C-fiber/A-fiber afferents was examined. Moreover, NGF-antibody (NGF-Ab) was previously administered into the hindlimb muscles of occluded rats to neutralize effects of NGF, and then SNA and BP responses to static muscle contraction and passive tendon stretch were examined. Muscle contraction was performed to evoke both mechano- and metabo-components of the EPR, and muscle stretch was employed to activate muscle mechanoreceptor. Also, to examine effects of NGF on the reflex responses to activation of muscle metaboreceptors, lactic acid was injected into the arterial blood supply of the hindlimb muscles after infusion of NGF-Ab in the hindlimb muscles of occluded rats.

The general hypotheses were that (1) protein expression of TRPV1, P2X3, and ASIC3 receptors in DRG and their responses with stimulation of those receptors are increased after femoral artery ligation and (2) NGF contributes to augmented reflex SNA and BP responses evoked by stimulation of metabolically sensitive muscle afferent nerves via enhancement of metabolic receptors expression such as ASIC3 in thin C-fiber afferent neurons.

## Altered Metabolic Receptors on Muscle Sensory Nerves after Femoral Artery Occlusion

A number of studies have been performed to examine how the muscle reflex mediated-SNA is engaged via sensory receptors mechanism. This review will focus on the roles of TRPV1, P2X, and ASICs receptors on muscle sensory nerves in mediating the exaggerated sympathetic response in hindlimb muscle ischemia seen in PAD patients. Note that those receptors to be studied appear at the peripheral terminals and the cell body of the sensory afferent neurons-DRG (Figure 1). Receptor activity of DRG cell bodies has been used frequently as a surrogate nerve ending receptor activity and physiology (Tsuzuki et al., 2003; Puntambekar et al., 2004). Especially, the whole cell patch clamp methods (Figure 1) are used to characterize the precise mechanisms by which those receptors mediate responses (Tsuzuki et al., 2003; Puntambekar et al., 2004).

### Transient receptor potential vanilloid type 1

It is known that TRPV1 receptor appears preferentially on metabolite sensitive Group III and IV sensory neurons (Ma, 2001). These receptors are located on afferents in a variety of tissues and mediate the effect of the vanilloid compound capsaicin (Caterina et al., 1997). When capsaicin is injected into the pulmonary circulation it activates C-fibers and evokes a pulmonary chemoreflex (Coleridge et al., 1989). The epicardial application of capsaicin stimulates cardiac TRPV1 receptors evoking a sympathoexcitatory reflex (Zahner et al., 2003). The competitive capsaicin antagonist capsazepine can reduce capsaicin-induced activation of the cloned non-selective cation channel TRPV1 (Caterina et al., 1997). Capsazepine also abolishes capsaicin-induced C-fiber activity both *in vitro* and *in vivo* (Fox et al., 1995; Lee et al., 1996). Although the endogenous TRPV1 ligand has not been determined, both the metabolic by-products accompanying the inflammatory process (lactic acid, H^+^) and inflammatory mediators themselves (histamine, serotonin, prostaglandin E2) have been identified as potential endogenous ligands for the C-fiber “capsaicin” receptor. Hydrogen ions (H^+^) in general and lactic acid in particular have been shown to activate C-fiber afferents similar to the effect seen with capsaicin (Stahl and Longhurst, 1992; Bevan and Geppetti, 1994; Hong et al., 1997). *In vitro* studies have demonstrated that H^+^ inhibits the binding of the capsaicin analog resiniferatoxin (RTX) to vanilloid receptors, a finding that was attributed to competition for the same binding site (Szallasi et al., 1995).

Activation of thin fiber muscle afferent nerves causes increases in BP and HR via a reflex muscle response (Kaufman et al., 1983, 1984; Kaufman and Forster, 1996b). When capsaicin is injected into the arterial supply of the dog hindlimb, BP rises by 20%, an effect is abolished by sectioning afferent nerves (Crayton et al., 1981). The muscle pressor response is likely to be due to the stimulation of both Group III and IV fibers since capsaicin stimulates 71% of Group IV and 26% of Group III dog hindlimb muscle afferent fibers (Kaufman et al., 1982). In a prior study, it has been observed that when capsaicin injected into the arterial supply of the hindlimb muscles of rats, BP increases and the effect is mediated via the TRPV1 receptors engagements on sensory afferents (Li et al., 2004a).

Consistent with those previous findings (Li et al., 2004a,b; Gao et al., 2006), it has been reported that TRPV1 can mediate SNA and pressor responses via a reflex mechanism (Xing et al., 2008a), and the responses are exaggerated by the femoral artery occlusion, suggesting that ischemia sensitizes TRPV1 receptors (Xing et al., 2008a). In addition, evidence from this prior study shows that: (1) arterial occlusion leads to upregulation of TRPV1 expression in the DRG neurons; and (2) the magnitude of capsaicin-evoked currents of the DRG neuron is greater in rats with the arterial occlusion. Thus, it is well reasoned that alterations in TRPV1 can contribute to enhanced sympathetically mediated vasoconstriction leading to reduced muscle blood flow in PAD.

**Figure 2:**
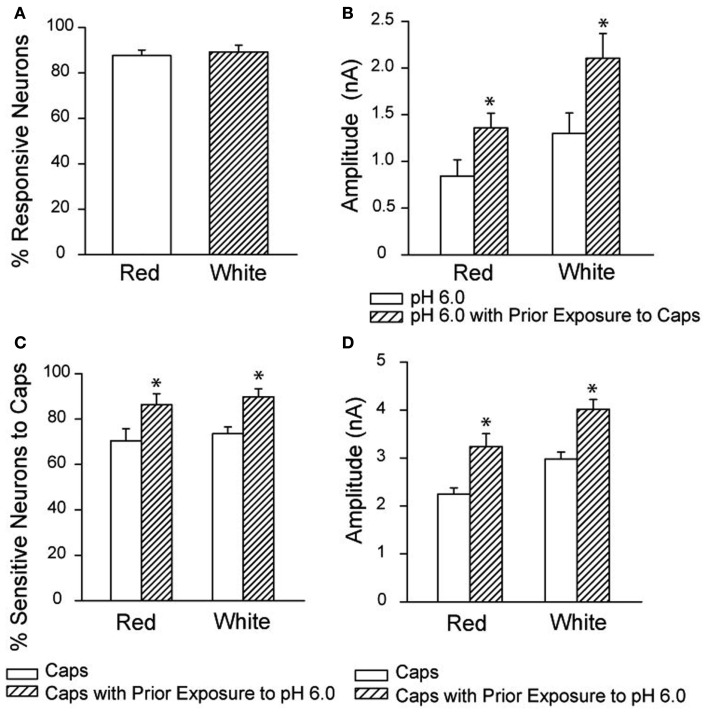
**Overlapping response to low pH and capsaicin in DRG neurons**. **(A)** The neurons that responded to pH 6.0 with a sustained inward current also responded to capsaicin. The overlap was similar for DRG neurons innervating the red and white muscle. **(B)** Prior exposure to capsaicin increased the amplitude of the proton-evoked currents in DRG neurons innervating the red and white muscles. *P < 0.05 compared with pH 6.0 without exposure to capsaicin. **(C,D)** Show that prior exposure to protons increased the proportion of DRG neurons responding to capsaicin, and increased the amplitude of the capsaicin-induced currents in the neurons. *P < 0.05 compared with capsaicin without exposure to pH 6.0. Reprinted from Xing et al. (2008b).

It should be noted that a prior study in the cat model indicates that blockade of TRPV1 does not attenuate the EPR (Kindig et al., 2005). In a rat model of femoral artery ligation, augmented BP response to static contraction of the hindlimb muscles was not seen to be significantly attenuated after blocking TRPV1 (Tsuchimochi et al., 2010a). It is speculated that TRPV1-induced reflex responses require H^+^ (lower pH) in the muscle interstitium. It has been reported that TRPV1 and ASIC play a coordinated role in the processing of muscle sensory signals (Gao et al., 2006). Likewise, ASIC responds to the accumulation of muscle metabolites (such as lactic acid/lowered pH, ATP, and inorganic phosphates) that are liberated by exercising muscles (Light et al., 2008). In situations without acidosis, the TRPV1 may not be effectively active. This hypothesis is supported by another work showing that receptors mediating protons and capsaicin responses coexist in the DRG neurons innervating muscle (Xing et al., 2008b). The responsiveness of acidosis and capsaicin is sensitized by each other, and amplitudes of inward currents responsive to protons and capsaicin are greater in the neurons innervating muscle comprised predominately of glycolytic fibers than that in those innervating muscle comprised predominately of oxidative fibers (Figure 2). This study also provides evidence at a cellular level that responsiveness of sensory neurons with nerve endings in different fiber types respond differently to a given level of metabolic stimulation. Note that the effect of fiber type composition on the cardiovascular responses evoked by static muscle contraction was studied previously (Wilson et al., 1995). Data of this study suggest that the reflex responses evoked by static contraction of oxidative muscle (red portion) are less, compared with the changes elicited by contraction of glycolytic muscle (white portion).

### P2X receptors

It has been reported that ATP and analogs of ATP stimulate and excite sensory afferent nerves *via* P2X purinoceptors on sensory nerves (Burnstock and Wood, 1996; Burnstock, 1999). Specifically, it has been shown that increased ATP in the hindlimb muscles elevates BP (Hanna et al., 2002; Li and Sinoway, 2002). In these studies, stimulation of ATP-sensitive P2X receptors in the hindlimb muscle increased BP. It has been confirmed that Group III and IV afferents are responsible for the increase in BP after arterial infusions of α, β-me ATP (Hanna and Kaufman, 2004). Also, it has been demonstrated that ATP enhances cardiovascular responses induced by stimulation of muscle mechanoreceptors *via* P2X receptors (Li and Sinoway, 2002).

**Figure 3:**
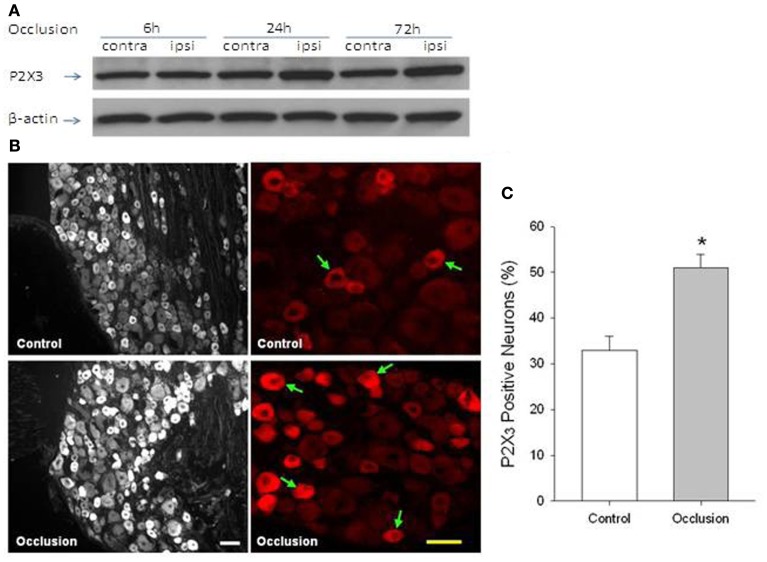
**(A)** Western blot assay was employed to examine P2X3 proteins in L4–L6 DRG at time of 6, 24, and 72 h following femoral artery occlusion. Representative bands of P2X3 expression show effects of femoral artery occlusion on P2X3 expression in dorsal root ganglion (DRG) neurons at different time courses. The ligation was performed on the one hindlimb [ipsilateral (ipsi)]. The sham-operated procedure was performed on the contralateral limb [contralateral (contra)] of the same rats, and this served as control. Bands of β-actin are used as control for an equal protein loading. **(B,C)** Fluorescence immunohistochemistry was employed to examine expression of P2X3 in DRG neurons of control limb and occluded limb. Representative photomicrographs and average data show that P2X3 appears within lumbar DRG neurons and that percentage of P2X3 positive neurons is greater in DRG neurons of 24 h of femoral artery occlusion (*n* = 6) than that in control (*n* = 6). Arrows indicate P2X3 positive cells. Scale bar = 50 μM. *P < 0.05, compared with control. Reprinted from Liu et al. (2011).

Data have been published demonstrating that interstitial ATP levels are elevated in active muscle of human subjects, dogs, and cats (Hellsten et al., 1998; Mo and Ballard, 2001; Li et al., 2003). It is anticipated that ischemic insult of the hindlimb muscles is likely to accumulate ATP to a larger degree, and thereby greater ATP levels can upregulate P2X receptors on thin fiber afferent nerves (Xu and Huang, 2002) and augment the P2X mediated-SNA response. On the basis of these data, it was hypothesized that femoral artery occlusion increases P2X3 receptors in DRG neurons which thereby leads to the enhanced reflex responses to stimulation of P2X3. Western blotting and immunohistochemistry were employed to examine P2X3 in DRG neurons of control rats and those with femoral artery occlusion. In order to determine P2X responsiveness, sympathetic, and cardiovascular responses to injections of α, β-me ATP into the arterial blood supply of the hindlimb muscles were further examined in both groups. Results of this study demonstrated that 24 and 72 h of femoral artery occlusion significantly elevated the protein levels of P2X3 in lumbar DRGs (Liu et al., 2011). Twenty hours following the ligation surgery, the level of P2X3 was 1.47-fold greater in occluded rats than in control animals. Fluorescence immunohistochemistry further confirmed that femoral occlusion increased P2X3 immunoreactivity in small- to medium-diameter of DRG neurons. These results are summarized in Figure 3. In addition, injection of α, β-me ATP into the arterial blood supply of the hindlimb muscles evoked greater increases in RSNA and MAP in occluded rats than in control rats (Liu et al., 2011). The findings of this study suggest that there is a close linkage in increased P2X3 receptors on afferent nerves and augmented sympathetic responsiveness to stimulation of muscle afferent nerves under conditions of femoral occlusion.

Notably, recent studies suggest that reactive oxidative stress (ROS) contributes to regulation of discharges of vagal lung thin afferent fiber nerves (Ruan et al., 2005, 2006). Also, it has been reported that an increase in muscle NADPH oxidase-derived ROS sensitizes the EPR in a decerebrate rat model (Wang et al., 2009). Likewise, a decrease in ROS can attenuate the reflex (Wang et al., 2009). Thus, it is speculated that ROS is engaged in augmented SNA and BP response during activation of the EPR in rats with femoral occlusion. Superoxide dismutases (SOD), a class of enzymes that catalyze the dismutation of superoxide into oxygen and hydrogen peroxide as considered an important antioxidant. In a published work, tempol, a mimic of SOD, was arterially injected into the hindlimb muscles of rats and results demonstrated that tempol attenuates BP response evoked by contraction of occluded hindlimb muscles, but the attenuation was not seen when contraction was induced in freely perfused control legs (McCord et al., 2011). A following study suggests that effects of tempol on the BP response during contraction are via ATP dependent potassium channels. However, a prior study suggests that ROS plays an important role in regulating discharges of vagal lung thin afferent fiber nerves via engagement of TRPV1 and P2X receptors (Ruan et al., 2005, 2006). In those experiments, the reflex pulmonary chemical response induced by a ROS stimulant hydrogen peroxide is attenuated by the prior application of i-RTX (TRPV1 antagonist) and PPADS (P2X antagonist; Ruan et al., 2005, 2006). Thus, it is likely that ROS can alter response of sensory nerves with activation of TRPV1 and P2X. Nevertheless, the augmented EPR is significantly attenuated after tempol is given to compensate SOD in occluded muscles of rats (McCord et al., 2011). If the levels of SOD in the hindlimb muscles are altered after femoral artery occlusion may be necessary to be examined given that anti-oxidation is likely to be beneficial to the augmented cardiovascular responses during exercise after femoral occlusion.

### Acid sensing ion channel

Lactic acid infused into the arterial supply of the hindlimb increases BP (Rotto et al., 1989; Sinoway et al., 1993; MacLean et al., 2000; Li et al., 2004a). A prior report demonstrates that H^+^ evokes reflex muscle responses via the stimulation of ASIC but not TRPV1 (Li et al., 2004a). Specifically, H^+^ evokes a pressor response that is not blocked by capsazepine but is attenuated by amiloride, an ASIC blocker. Of note, with pretreatment of RTX to destroy muscle afferents containing TRPV1 receptors, both capsaicin and H^+^ responses are blunted (Li et al., 2004a). This suggests that ASIC are likely to be frequently found on afferents containing TRPV1 receptors. Another report suggests that TRPV1 and ASIC play a coordinated and interactive role in the processing of muscle afferent response to acid phosphate (Gao et al., 2006). In this report, it has been observed that simultaneous attenuation of TRPV1 and ASIC blunts acid phosphate-induced pressor response to a larger degree than when the respective blockers are given separately (Gao et al., 2006).

Furthermore, a study has used a rat model of femoral artery ligation to demonstrate that the cardiovascular responses to static contraction are amplified in occluded rats compared with control rats (Liu et al., 2010). A recent study has further shown that arterial injection of a specific ASIC3 blocker markedly attenuates the reflex pressor response to muscle contraction in the rats with a ligated femoral artery, but has only modest effects in the rats with freely perfused hindlimbs (Tsuchimochi et al., 2011). Notably, ASIC3 expression is upregulated in DRG neurons innervating the hindlimb muscles with the occluded femoral artery (Liu et al., 2010). Additionally, injecting lactic acid into the arterial blood supply of hindlimb muscles to stimulate ASIC3 of muscle afferent nerves increases SNA and BP to a greater degree in occluded rats (Liu et al., 2010).

**Figure 4:**
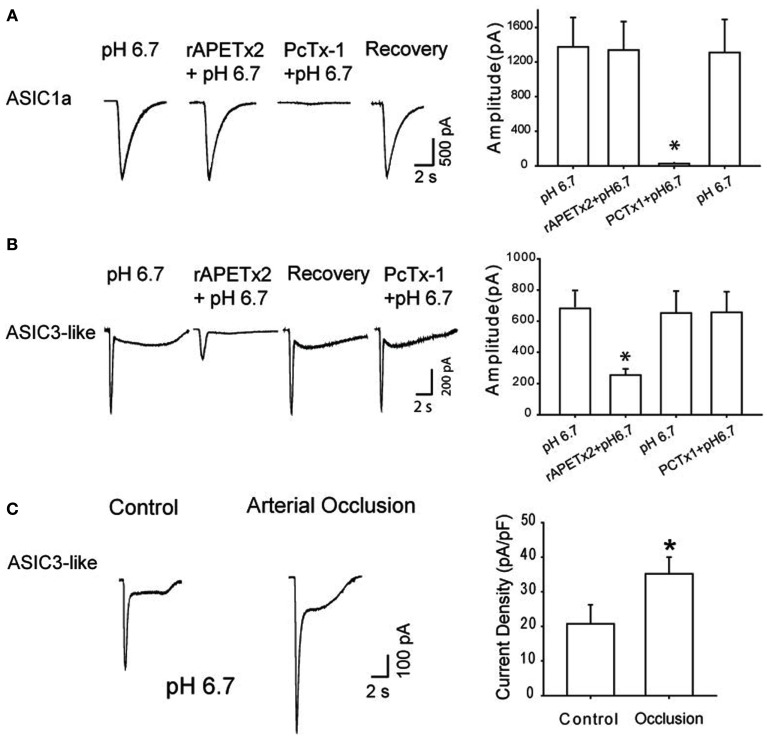
**(A,B)** Toxins PcTx1 and rAPETx2 were used as antagonists to specifically block ASIC1a and to ASIC3-like currents evoked by pH 6.7 in DRG neurons innervating the hindlimb muscles. Toxin PcTx1 (20 nM) significantly inhibited current responses induced by pH 6.7 in DRG neurons exhibiting ASIC1a currents. However, rAPETx2 had no significant effects on this type of current in DRG neurons. Similarly, a prior application of rAPETx2 (1 μM) significantly attenuated peak amplitudes of currents evoked by pH 6.7 in DRG neurons that displayed ASIC3-like currents. PcTx1 had negligible effects on this type of currents. *P < 0.05 vs. pH 6.7 alone. Note that the inhibitory effects of PcTx1 on ASIC1a currents and rAPETx2 on ASIC3-like currents were both reversible. **(C)** Original traces and averaged data show effects of femoral arterial occlusion on ASIC3-like currents response to pH 6.7. Current density was used to analyze response of ASIC3-like currents. Twenty-four hours of arterial occlusion induced a larger current density compared with control. *P < 0.05 vs. control. Reprinted from Xing et al. (2012).

Among ASICs, ASIC3 is found predominantly on sensory neurons and maintains functional channels in response to proton concentration fluctuation (Waldmann et al., 1997a,b, 1999; Light et al., 2008). The pH range required to activate ASIC3 is ~6.5–7.0 (Deval et al., 2008, 2011), which is close to what is observed in exercising muscle and/or moderately ischemic tissues (Rotto et al., 1989; MacLean et al., 1998, 2000; Yagi et al., 2006). Thus, in a prior study whole cell patch clamp methods were employed and acid-induced current with ASIC3 activation in DRG neurons of control rats and rats with 24 h of femoral artery occlusion were characterized to examine the mechanisms by which ASIC3 is engaged in femoral occlusion-augmented responses (Xing et al., 2012). The data of this study indicate that in DRG neurons with nerve endings in the hindlimb muscles, ASIC3-containing channels represent the majority of acid-induced currents elicited by moderate external acidosis in a range that is relevant to exercising muscle and/or hindlimb ischemia. Additionally, a greater current response with activation of ASIC3 is observed as the arterial blood supply to the hindlimb is deficient under ischemic conditions (Figure 4). Note that the size of DRG neurons that have ASIC3-like currents is typically small to large, and the size distribution is similar in control and occluded animals. Also, the percentage of DRG neurons with pH 6.7-triggered action potential is greater in occluded rats than in control rats, suggesting that femoral occlusion increases the probability of sensory neurons to evoke neuronal activities (Xing et al., 2012). The results of immunohistochemical experiments further suggest that ASIC3 appears in both C- and A-fibers of DRG neurons, and that femoral artery occlusion largely increases expression of ASIC3 in DRG neurons that project C-fiber afferents (Xing et al., 2012).

### Other receptors

In addition to TRPV1, P2X3, and ASIC3, it needs to point out that other muscle afferents’ receptors including μ-opioid and thromboxane (TP) receptors are engaged in processing chronic ischemia of the hindlimb muscles (Tsuchimochi et al., 2010b; Leal et al., 2011). Stimulating peripheral μ-opioid receptors using DAMGO significantly attenuates the pressor responses to static contraction in rats with 72 h of femoral artery ligation compared with freely perfused rats (Tsuchimochi et al., 2010b). Also, this study demonstrates that the inhibitory effect of DAMGO can be prevented by the injection of naloxone, an opioid blocker. In another published work, arterial injection of daltroban, a TP receptor antagonist, into the hindlimb muscles has been shown to attenuate the cardiovascular responses to static contraction and tendon stretch in 72 h ligated rats, suggesting that TP receptor contributes to the EPR in PAD (Leal et al., 2011).

## Role for NGF in Regulating Responses of Afferent Metabolic Receptors and Exercise Pressor Reflex in Ischemic Muscle

Prior studies demonstrated that femoral artery occlusion elevates the levels of NGF in the hindlimb muscles and DRG neurons of rats (Emanueli et al., 2002; Xing et al., 2009). NGF can induce expression of TRPV1, P2X3, and ASIC3 receptors in the DRG neurons (Ramer et al., 2001; Mamet et al., 2003). In addition, NGF can change the neuronal phenotype such as capsaicin-insensitive sensory neurons (Hunter et al., 2000), which possibly alters afferent mediated response in the processing of sensory signals. Therefore, in a series of experiments, the role for NGF in regulating those metabolically sensitive receptors in muscle afferent nerves was examined. Also, NGF-Ab was previously administered into the hindlimb muscles of occluded rats to neutralize effects of NGF, and then SNA and BP responses to static muscle contraction and passive tendon stretch were examined. Muscle contraction is performed to evoke both mechano- and metabo-components of the EPR, and muscle stretch is employed to activate muscle mechanoreceptor. Also, to determine effects of NGF on the reflex responses to activation of muscle metaboreceptors, SNA and BP responses to lactic acid injected into the arterial blood supply of the hindlimb muscles were examined after infusion of NGF-Ab in the hindlimb muscles of occluded rats.

Thin fiber afferent nerves (neurons) are distinct as IB_4_-negative and IB_4_-positive because of their distinct neurochemical characteristics and neurotrophic factor responsiveness. The IB_4_-negative neurons express trkA receptors for NGF, depend on NGF for survival during postnatal development, and contain neuropeptides such as calcitonin gene-related peptide and substance P (Averill et al., 1995; Bennett et al., 1996, 1998; Molliver et al., 1997). The IB_4_-positive neurons express receptors for glial cell line-derived neurotrophic factor (GDNF), depend on GDNF for survival during postnatal development, and are relatively “peptide poor” but express a surface carbohydrate group that binds IB_4_ (Averill et al., 1995; Bennett et al., 1996, 1998; Molliver et al., 1997).

The data show that arterial occlusion augments responses with activation of metabolite sensitive TRPV1 receptors in IB_4_-positive, and -negative DRG neurons (Xing et al., 2009). Additional experiment showed that increased NGF in the muscles and in the culture dish containing DRG neurons amplifies the magnitude of TRPV1 response to capsaicin in IB_4_-negative DRG neurons but not in IB_4_-positive DRG neurons. These findings suggest that NGF plays a role in augmented TRPV1 responses in the processing of muscle ischemia or vascular insufficiency induced by the femoral artery occlusion (Xing et al., 2009). The data further suggest that a selective subpopulation of the afferent neurons is engaged in NGF augmented TRPV1 response. Thus, evidence of this study provides strong support for the proposition that NGF regulation in muscle metabolic changes associated TRPV1 receptors contributes to augmented sympathetic activity and may lead to a reduction in exercise capacity seen in PAD.

Furthermore, infusion of NGF into the hindlimb of healthy rats through a micro-osmotic pump induces 1.39-fold increases in P2X3 protein of the DRGs of the infused leg as compared to the control leg (Liu et al., 2011). Also, NGF infused into the hindlimb significantly enhanced the pressor response to arterial injection of α, β-me ATP. On the other hand, blocking NGF attenuated exaggeration of the reflex response induced by α, β-methylene ATP in occluded rats (Liu et al., 2011). These findings suggest that NGF is closely related to upregulation of P2X3 expression in DRG neurons and to augmentation in the SAN and BP responses to activation of P2X3 as the hindlimb vascular insufficiency occurs.

Finally, the role of NGF in regulating the enhanced sympathetic responsiveness during stimulation of primary muscle afferent nerves was examined as the blood flow directed to hindlimb muscles is insufficient following femoral artery ligation. In the first set of experiments, NGF-Ab was previously injected into the hindlimb muscles to block effects of NGF that were induced by femoral occlusion. Then, mechano- and/or metabo-sensitive afferents nerves were activated by three interventions, namely muscle contraction, tendon stretch, and stimulation of ASICs using lactic acid. The data demonstrate that NGF neutralization significantly attenuates femoral occlusion-augmented reflex sympathetic and BP responses evoked by static contraction and lactic acid, but not by muscle stretch. Given that administration of NGF-Ab into the hindlimb muscles significantly attenuates occlusion-enhanced protein levels of ASIC3 in DRG tissues, these results suggest that NGF that is increased in sensory nerves of occluded limbs contributes to augmented reflex SNA and BP responses to stimulation of chemically, but not mechanically sensitive muscle afferent nerves. Also, this study has examined whether NGF is engaged in the role of sensory nerves’ ASIC3 in augmented responses evoked by the hindlimb vascular insufficiency. In the second set of experiments, effects of NGF on expression of ASIC3 in DRG neurons that project thin C-fiber and A-fiber were examined. NGF infused into the hindlimb muscles significantly increases the protein levels of ASIC3 in DRG and selectively increase expression of ASIC3 in DRG neurons that project C-fiber afferents. Thus, these data support the hypothesis that NGF plays a role in exaggeration of the muscle metaboreflex via enhancement of ASIC3 expression in thin C-fiber afferent neurons.

## Summary

**Figure 5:**
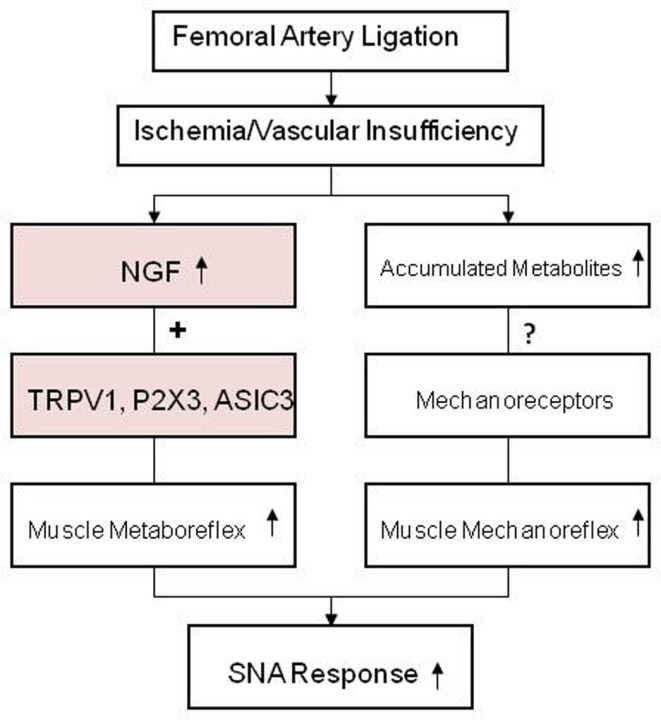
**The schematic diagram summarizes the regulatory mechanisms of the exaggerated sympathetic response to activation of the muscle metabo- and mechano-sensitive afferent nerves after femoral artery ligation**.

Peripheral artery disease affects life styles in 20% of adults who are older than 65 years. The narrowing of blood vessels of the lower limbs, mainly due to atherosclerotic vascular disease, is a main cause of PAD. Intermittent claudication is the most common symptom of this disease and it regularly occurs during exercise/physical activity but is relieved promptly by rest. It is well known that the sympathetic nerve system plays an important role in regulating blood flow directed to skeletal muscle tissues during exercise. Thus, the goal of those studies was to examine the role of metabolite sensitive receptors on muscle afferent neurons in the responses of SNA after limiting blood flow to hindlimb muscle, as seen in PAD. Currently, several studies using a rat model of femoral artery ligation show that sympathetic responses of the EPR engagement are exaggerated as seen in PAD patients. Findings of the completed studies also suggest that enhanced protein levels of TRPV1, P2X3, and ASIC3 in muscle afferent nerves and amplified responses of those receptors contributes to the exaggerated reflex sympathetic and pressor responses to their individual receptor stimulus (Figure 5). The findings further suggest that NGF is likely responsible for enhanced TRPV1, P2X3, and ASIC3 and plays a role in modulating the metaboreceptor component of the EPR in the hindlimb muscle ischemia (Figure 5). Lactic acid and ATP are the major muscle by-products in exercising muscles and TRPV1, P2X3, and ASIC3 receptors are sensitive to those individual metabolites or combined metabolites. Current data presented here provide evidence that alteration in chemically sensitive receptors TRPV1, P2X3, and ASIC3 in primary afferent neurons innervating ischemic muscles plays an important role in the development of the exaggerated reflex sympathetic response, likely leading to worsening exercise capacity in patients with PAD. Also, NGF that increased in sensory nerves is engaged in abnormal responses of those metabolic receptors. A mechanism responsible for the augmented sympathetic response to the muscle mechanoreflex needs to be determined in the future. It is speculated that muscle metabolites are accumulated to a greater degree in ischemic muscles of PAD, which can sensitize mechanically sensitive muscle afferent nerves and enhance sympathetic response during activation of muscle mechanoreflex (Figure 5).

## Conflict of Interest Statement

The authors declare that the research was conducted in the absence of any commercial or financial relationships that could be construed as a potential conflict of interest.
